# Topical Antibiotic Treatment in Dermatology

**DOI:** 10.3390/antibiotics12020188

**Published:** 2023-01-17

**Authors:** Matthew Dallo, Kavina Patel, Adelaide A. Hebert

**Affiliations:** 1 Department of Dermatology, The University of Texas Health Houston, Houston, TX 77030, USA; 2 Department of Dermatology and Pediatrics, The University of Texas Health Houston, Houston, TX 77030, USA

**Keywords:** topical antibiotics, skin infections, dermatology, antimicrobials, inflammatory dermatoses

## Abstract

Many indications in dermatology can be effectively managed with topical antibiotics, including acne vulgaris, wound infections, secondarily infected dermatitis, and impetigo. Dermatologists must be familiar with the wide spectrum of topical antibiotics available, including indications, mechanisms of action, adverse events, and spectra of activity. Dermatologists must also keep antibiotic resistance in mind when utilizing these medications. Due to the widespread use of topical antibiotics and their importance in dermatology, a literature review was performed using a systematic search of PubMed and Google Scholar with the terms topical antibiotics, skin infections, dermatology, antimicrobials, and inflammatory dermatoses to identify English-language articles published between 1965–2022 from any country. Relevant publications were manually reviewed for additional content. The following literature review will summarize the common topical antibiotics used in dermatology.

## 1. Introduction

Topical antibiotics play a key role in dermatology and have several uses, including mild-to-moderate acne vulgaris, secondarily infected dermatitis, rosacea, treatment and prevention of wound infections, and impetigo [1]. Topical formulations allow for targeted delivery of the active ingredient to the site of dermatologic concern, along with higher concentration of the active ingredient, with avoidance of systemic adverse events or toxicity. Other benefits include reduced disruption of the intestinal microbial flora, low cost, and ease of administration. Disadvantages of topical therapies include the emergence of bacterial resistance, ability to only target superficial wounds, and the possibility of inducing contact dermatitis. These medications are chosen based on the micro-organism that needs to be eradicated and the location of the dermatologic concern. With the increase in prevalence of bacterial resistance, dermatologists should take extra precautions when prescribing these medications. Dermatologists must be familiar with the various topical antibiotics in the realm of dermatology, and their indications, mechanisms of action, and efficacy, to easily incorporate these medications into their routine practice and improve patient care.

## 2. Methods

The aim of this literature review is to familiarize dermatologists with the different topical antibiotics in dermatology. We performed Internet-based searches of PubMed and Google Scholar using the keywords topical antibiotics, skin infections, dermatology, antimicrobials, and inflammatory dermatoses, in various combinations to identify English-language articles published between 1965–2022 from any country. Relevant publications were manually reviewed for additional content. This literature review will present the various topical antibiotics in alphabetical order.

## 3. Discussion

### 3.1. Amikacin

Amikacin is a semi-synthetic aminoglycoside antibiotic that is derived from kanamycin A. Amikacin’s mechanism of action relies on its ability to bind to bacterial 30S ribosomal subunits and interfere with mRNA binding and tRNA acceptor sites, inhibiting bacterial growth. Amikacin exerts activity against Gram-negative bacteria such as *Pseudomonas aeruginosa*, *Acinetobacter*, and *Enterobacter*. Amikacin is primarily available as an intravenous, intramuscular, or inhalation formulation. Topical formulations are available in certain countries, but not in the United States, and has not been approved for cutaneous infections. Topical preparations include a 5% gel or cream, which has been used for Gram-negative folliculitis (hot-tub folliculitis) [2] and has been studied for the treatment of peristomal dermatitis [3].

### 3.2. Azelaic Acid

Azelaic acid is a naturally occurring and nontoxic dicarboxylic acid that possesses significant properties as a therapeutic agent [4]. The dicarboxylic acid molecule first received attention as an inducer of hypopigmentation via competitive inhibition of tyrosinase. This inspired the use of azelaic acid as a topical treatment for hyperpigmented disorders. This investigation further demonstrated azelaic acid’s unique anti-inflammatory and antibacterial properties.

Azelaic acid functions as a reversible inhibitor of tyrosinase and other oxidoreductases, inhibiting mitochondrial respiration [4]. This medication is also shown to inhibit anaerobic glycolysis. Due to its distinct mechanism of action, azelaic acid has been shown to have both in vitro and in vivo activity against aerobic and anaerobic micro-organisms [5]. This makes azelaic acid an ideal agent for the treatment of *Cutibacterium acnes* (*C. acnes*), *S. aureus*, and *S. epidermidis* [4]. Besides the antimicrobial properties of azelaic acid, its anti-inflammatory effects are not only a consequence of its ability to mitigate disease but due to a reduction in the production of pro-inflammatory factors and reactive oxygen species [4]. Azelaic acid has also been shown to influence the differentiation of human keratinocytes by reducing the synthesis of keratinocyte precursors. This reduction causes azelaic acid to act as a mild anti-keratinizing agent and alter the phases of epidermal differentiation [4]. This helps reduce the blockage of follicular ducts.

Azelaic acid was first approved as a 15% gel in 2002 for the treatment of inflammatory papules and pustules of mild-to-moderate rosacea [6]. The gel is typically applied twice daily for a total of 12 weeks. Clinical studies have shown that the monotherapeutic use of the 15% gel and 20% cream formulations are efficacious in the treatment of papulo-pustular rosacea, but the 15% gel is the only formulation currently approved for this indication [6]. Azelaic acid has also been used in various formulations to treat lentigo maligna, malignant melanoma, and perioral dermatitis. Azelaic acid 20% cream is used for the treatment of mild-to-moderate inflammatory acne in adults and children 12 years and older, and is sometimes used as an off-label treatment for melasma [5].

Adverse events of azelaic acid include erythema, pruritus, stinging, burning, dryness, and tingling at the affected site. Hypersensitivity reactions (e.g., angioedema, dyspnea, eye swelling, facial swelling, and urticaria) have been reported, along with hypopigmentation. Patients should discontinue medication use if any of the above symptoms develop.

### 3.3. Benzoyl Peroxide

Benzoyl peroxide is a topical agent derived from coal tar and an FDA-approved medication for the treatment of mild-to-moderate acne vulgaris. Topical benzoyl peroxide demonstrates bactericidal activity against *C. acnes* on the skin and within hair follicles. Benzoyl peroxide also possesses antibacterial activity against Gram-positive bacteria and fungi, as demonstrated in an in vitro study evaluating the microbicidal activity of benzoyl peroxide against *S. aureus*, *S. epidermidis*, *Candida albicans*, *Malassezia furfur*, *Malassezia restricta*, and *Malassezia globose* [7]. The study showed that benzoyl peroxide had rapid and potent bactericidal activity against the organisms, but lack of efficacy against Gram-negative bacteria [7]. Benzoyl peroxide functions in the pilosebaceous follicles. When applied and absorbed by the skin, benzoyl peroxide is converted to benzoic acid. Benzoic acid is than metabolized by cysteine in the skin, resulting in the production of free-radical oxygen species [8]. These free radicals cause oxidation of bacterial proteins in the cell membrane, leading to bacterial destruction [7,8,9]. Due to benzoyl peroxide’s complex mechanism of action, there are no reports of bacterial resistance to the topical agent.

Benzoyl peroxide is available as both over-the-counter and prescription formulations in concentrations of 2.5%, 5%, and 10%. The preparations that are available include lotions, creams, gels, foams, and solutions. Benzoyl peroxide has mild sebostatic and keratolytic effects, and is most effective when combined with other acne vulgaris therapies. Common combination preparations include erythromycin or clindamycin, which are applied twice daily, or preparations with adapalene, applied once daily [8]. Off-label indications of benzoyl peroxide include inflammatory forms of rosacea, folliculitis, pseudo-folliculitis, progressive macular hypomelanosis, pressure ulcers, and pitted keratolysis [8]. The adverse events of benzoyl peroxide are limited, mostly compromising skin irritation.

### 3.4. Clindamycin

Clindamycin is a semi-synthetic lincosamide antibiotic that has replaced lincomycin, a naturally occurring lincosamide that was first isolated from the soil bacterium *Streptomyces lincolnensis* in Lincoln, Nebraska. This replacement by clindamycin is due to its higher efficacy and wider range of susceptible organisms compared to lincomycin [10]. Clindamycin functions by inhibiting bacterial protein synthesis by binding to 23S rRNA of the 50S subunit of the bacterial ribosome. This causes a disruption to the assembly of the ribosome and the translation process, halting bacterial growth.

Clindamycin comes in oral and parenteral formulations, but as a topical agent it is typically formulated as either a gel, lotion, solution, or foam. Topical clindamycin products in the US have a 1–2% concentration and can come in two combination products: clindamycin phosphate 1.2%/tretinoin 0.025% gel and benzoyl peroxide 5%/clindamycin 1% gel/lotion. The combination gel formulation of tretinoin 0.025%/clindamycin 1% is currently undergoing investigation.

Clindamycin has a number of indications, but when used topically it is indicated for the treatment of acne vulgaris, targeting the Gram-positive anaerobic bacterium *Cutibacterium acnes* (*C. acnes*). The beneficial effects of clindamycin are due both to non-antimicrobial and antimicrobial effects. Examples of non-antimicrobial effects include inhibition of the production of polymorphonuclear chemotactic factors, lipase, and neutrophilic chemotactic factors, while the latter is due to suppression of the growth of *C. acnes* [11].

The use of topical clindamycin demonstrated a significant reduction in lesion count and patient assessments when compared to placebo in a number of clinical trials investigating acne vulgaris [11]. In a randomized double-blinded study performed by Braathen et al., topical clindamycin phosphate 1% b.i.d., oral tetracycline 500 mg b.i.d., and placebo were compared for efficacy in the treatment of acne vulgaris. Topical clindamycin phosphate demonstrated a highly significant decrease of inflammatory lesions of 72%, while tetracycline and placebo demonstrated a 57% and 12% decrease, respectively [12]. Another study performed by Resh et al. demonstrated that the use of topical clindamycin phosphate showed a dramatic suppression of *C. acnes* growth in patients with acne vulgaris in comparison to patients using topical erythromycin or tetracycline [13]. When compared to oral minocycline, topical clindamycin is just as effective as an alternative in the treatment of moderate-to-severe facial acne vulgaris [14].

Topical clindamycin 1% solution has been used off-label for the treatment of hidradenitis suppurativa. A 12-week randomized placebo-controlled trial of subjects with Hurley stage I or II disease demonstrated reduced pustules, but no effect on inflammatory nodules and abscesses when compared to placebo [15,16]. When compared to systemic tetracycline and topical clindamycin phosphate 1% solution, there was no significant difference between the two types of treatment in patients with mild-to-moderate disease [16,17]. Despite being well tolerated, topical clindamycin was shown to increase rates of *S. aureus* resistance in patients with hidradenitis suppurativa, highlighting the importance of stewardship in antibiotic therapy for patients with hidradenitis suppurativa [18].

The widespread use of clindamycin as a topical agent for the treatment of acne vulgaris has slowly led to the development of a macrolide–clindamycin-resistant *C. acnes* strain. This increase in resistance has been noted throughout the world, but a recent analysis in Japan has noted an increase from 20.3% in 2009 to 2010 to 44.1% in 2016 to 2017 [19]. The mechanisms of resistance to macrolide–clindamycin *C. acnes* strains are through 23S rRNA mutations and methylation of 23S rRNA by a ribosomal methylase gene *erm*(X) [19]. The development of 23S rRNA mutations is predominately due to the overuse of topical clindamycin commonly seen in acne patients. The presence of the *erm*(X) gene results from horizontal transfer among *C. acne* strains [19]. In a study performed in Japan by Aoki et al., it was shown that strains carrying the *erm*(X) gene have increased six-fold from 2010 and 2013 to 2015, leading to an increased prevalence of *C. acnes*-resistant strains [19]. In order to prevent the further emergence of multi-drug-resistant *C. acnes* strains, monitoring should be performed to prevent the threat to the antimicrobial therapy for acnes vulgaris.

Inducible resistance to clindamycin has also been noted in *S. aureus* in other parts of the world, such as in Africa, with a prevalence ranging from 2.9% to 44.0% [20]. This increasing resistance is largely due to the overutilization of antibiotics and underutilization of tests to identify antimicrobial resistance. Many of the *S. aureus* isolates produce resistance genes such as erm (A, B, C, E), msrA, mphC, and lnuA genes, with the most prevalent being erm genes [20]. This emerging resistance can lead to future health hazards worldwide, as clindamycin is relied upon for the treatment of a number of conditions, including *S. aureus*-associated pneumonia, and skin and soft-tissue infections. Genotypic detection of resistance genes can lead to a reduction in the utilization of clindamycin and possibly a decrease in *S. aureus*-inducible clindamycin resistance worldwide [20].

The adverse events associated with topical clindamycin usually involve local adverse reactions, such as erythema, desquamation, dryness, and burning [11,21]. There have been two published cases of pseudomembranous or *Clostridium difficile* colitis associated with topical clindamycin use, along with cases of contact dermatitis and diarrhea [11]. The two published cases of pseudomembranous colitis involved a 24-year-old female with no significant medical history and a 42-year-old female with uncomplicated Down syndrome. They were both prescribed topical clindamycin for the treatment of acne vulgaris [11,22,23]. Despite these serious adverse events, topical clindamycin has enjoyed widespread use over the past 30 years due to its efficacy and overall safety profile.

### 3.5. Dapsone

Dapsone is a sulfone drug that is used to treat a variety of cutaneous conditions: acne vulgaris, bullous systemic lupus erythematosus, dermatitis herpetiformis, pemphigus vulgaris, and Hansen’s disease. Dapsone is shown to have both anti-inflammatory immunosuppressive and antibacterial properties. Dapsone acts against bacteria and protozoa with similar characteristics to that of sulfonamides by inhibiting the synthesis of dihydrofolic acid. In relation to the drug’s anti-inflammatory action, the mechanism is not similar to its antimicrobial effect. Dapsone inhibits neutrophil chemotaxis and down-regulates interleukin-8, which plays a role in neutrophil-mediated inflammation [24]. Studies have shown that dapsone inhibits the complement activation alternative pathway in vitro and the myeloperoxidase enzyme system in neutrophils [24]. These properties make topical dapsone an effective treatment for its main indication of acne vulgaris.

Topical dapsone was developed as an alternative for the use of oral dapsone in acne vulgaris in order to minimize its negative systemic effects. Two pivotal phase III clinical trials showed a reduction in inflammatory and noninflammatory lesion counts compared to placebo in patients aged twelve and up with moderate acne vulgaris using topical dapsone 5% gel [25,26]. The topical dapsone 5% gel formulation was eventually approved by the US Food and Drug Administration (FDA) in 2008 based on a number of randomized trials; however, these trials lacked data on the efficacy and safety profile of the drug in patients under 12 years of age [24]. This formulation was later followed by the development of topical dapsone 7.5% gel, which was approved by the FDA in 2017. The advantage of this formulation is its once-a-day application, which improves patient compliance despite having the same safety and efficacy profile of the topical dapsone 5% gel formulation, which requires twice-a-day dosing [24].

The adverse events associated with topical dapsone are typically mild and usually involve dryness, peeling, and a burning sensation applied to the affected area. The combination of topical dapsone and benzoyl peroxide is not recommended due to the potential for yellow or orange discoloration on both skin and facial hairs. Discoloration typically resolves in 1 to 8 weeks after discontinuation. Patients with glucose-6-phosphate dehydrogenase deficiency (G6PD) have been observed to develop hemolytic anemia when using dapsone 5% gel twice daily. Methemoglobinemia is a rare potential complication of topical dapsone use, especially in patients with G6PD or congenital or idiopathic methemoglobinemia [27].

### 3.6. Fusidic Acid

Fusidic acid, while not available in the USA, is a bactericidal antibiotic that is available as an oral, injectable, and 2% topical cream or ointment. This medication is isolated from the fermentation broth of the fungus *Fusidium coccineum* and acts by interfering with bacterial protein synthesis, primarily preventing the translocation of elongation factor G (EF-G) from the ribosome [28]. The unique chemical structure of fusidic acid, which involves the presence of a steroid-like structure, classifies this medication as a steroidal antibiotic. Despite structural similarities to steroids, fusidic acid does not possess any steroid activity, but the structure is thought to be responsible for steroid-like high penetration and for the fact that no cross-allergy or cross-resistance has been seen with routine use [29]. The spectrum of activity of fusidic acid is primarily against Gram-positive bacteria (e.g., MSSA, MRSA, coagulase-negative *S. aureus*, Enterococci, *S. pyogenes*, etc.) with limited activity against Gram-negative bacteria due to its large size and lipophilicity, preventing transport through the porins in the outer membrane of these bacteria [30].

The clinical indications for fusidic acid in dermatology is the treatment of mild-to-moderate severe skin and soft-tissue infections, such as impetigo, folliculitis, erythrasma, furunculosis, and mild-to-moderate atopic dermatitis with the presence of secondary infection [29]. The benefits of topical fusidic acid in cutaneous conditions compared to that of other topical antibiotics, such as gentamicin or mupirocin, is that fusidic acid reaches higher antimicrobial concentration at deep skin layers after topical application either on intact or damaged skin [29,31]. Fusidic acid has also been shown to have anti-inflammatory activity due to the suppression of cytokine signaling. Additionally, fusidic acid presents a lower risk of resistance even in MRSA strains, a common pathogen in skin infections and infected atopic dermatitis. Combination formulations of fusidic acid with 1% hydrocortisone or 0.1% betamethasone has been shown to achieve excellent results in infected eczema [32]. These lipid-rich formulations help to create an extra moisturizing effect on the skin, and the development of resistance to fusidic acid can be further minimized by restricting therapy to no more than 14 days at a time [32].

The utility of topical fusidic acid in the treatment of hidradenitis suppurativa (HS) is limited to case reports and a single prospective cohort study. In the study, patients with Hurley stage I axillary HS received fusidic acid 2% ointment, which was applied three times a day after washing with antibacterial soap. The data demonstrated patients with complete healing within 4 weeks of treatment [33]. The data, however, have not been replicated elsewhere, and further studies to evaluate the efficacy of topical fusidic acid cream or ointment are warranted to further investigate its effectiveness in patients with hidradenitis suppurativa.

Adverse events during treatment are uncommon, and are limited to reactions to components of the vehicle, which can lead to irritant contact dermatitis or mild allergies [34].

### 3.7. Gentamicin

Topical gentamicin is a bacterial aminoglycoside that was discovered and isolated from *Micromonospora purpurea* in 1963 [35]. This antibiotic functions by binding to the 30S ribosomal subunit of the bacterial ribosome and ceasing protein synthesis on the target pathogenic bacteria. The spectrum of activity includes Gram-positive and Gram-negative aerobes. Topical gentamicin comes as a 0.1% cream or ointment and is indicated for primary skin infections (impetigo, superficial folliculitis, ecthyma, furunculosis, and pyoderma gangrenosum) and secondary skin infections (infectious eczematoid dermatitis, pustular acne, pustular psoriasis, infected seborrheic dermatitis, and infected contact dermatitis). This topical formulation can be used in both children and adults.

### 3.8. Metronidazole

Metronidazole is a commonly used antibiotic that belongs to the nitroimidazole class of antibiotics. Metronidazole comes in oral, intravenous, and topical formulations. The indications for topical metronidazole in cutaneous conditions focus on rosacea, while oral formulations can help treat severe forms of hidradenitis suppurativa [36]. The mechanism of action of metronidazole has not been fully established, but it is suggested that an intermediate in the reduction of metronidazole, which can only be made by anaerobic bacteria and protozoa, binds to DNA and the electron-transport proteins of organisms, blocking nucleic acid synthesis.

The mechanism by which metronidazole reduces the inflammatory component of rosacea has not been clearly established. Several in vitro studies have shown that the drug reduces tissue injury by inhibiting neutrophil-generated inflammatory mediators and reactive oxygen species generated by neutrophils [37]. Metronidazole is inactive against *Demodex folliculorum*, a parasitic mite found in the follicles of patients with rosacea. Similarly, application of metronidazole cream has shown to have no effect on staphylococci, streptococci, cutibacteria, or anaerobic cocci in patients with rosacea. This demonstrates that the action of metronidazole in patients with rosacea depends greatly on its anti-inflammatory properties rather than its ability to suppress skin bacteria [37].

Metronidazole comes as a gel, lotion, or cream formation at 0.75% which is to be applied twice a day, or cream or gel formulation at 1% applied daily for the management of rosacea. It has been shown that topical metronidazole decreases erythema, pustules and papules, but has no effect on telangiectasias. In a double-blind clinical trial, metronidazole resulted in a 65% reduction of papules and pustules in comparison to a 15% reduction by placebo [38]. In another double-blind trial conducted by Nielson lasting two months, 65% of the subjects had satisfactory clinical responses compared to 20% of those on vehicle [39].

The most common adverse events of topical metronidazole include erythema, irritation, dry, scaly, or itchy skin, or burning or stinging. Numbness or tingling sensation after administration can result and requires prompt discontinuation and evaluation by a clinician.

### 3.9. Minocycline

Topical minocycline is a semi-synthetic, second-generation, tetracycline-class drug available as a foam in a 1.5% or 4% preparation. Topical minocycline was developed to minimize systemic absorption and toxicity associated with oral minocycline use. Topical minocycline 1.5% is indicated for the treatment of moderate-to-severe papulopustular rosacea in adults, while topical minocycline 4% is indicated for the treatment of inflammatory lesions of non-nodular moderate-to severe acne vulgaris in patients 9 years of age and older [40,41,42]. Topical minocycline 4% foam contains a high lipid content, which allows the drug to be delivered through sebum to the affected pilosebaceous units without penetrating the dermis [43]. Topical minocycline has a wide range of antibacterial properties against Gram-positive and Gram-negative bacteria. In vitro studies have shown topical minocycline to have bacteriostatic activity against *C. acnes*. Topical minocycline induces this effect by binding to 16s rRNA and overlapping the anticodon stem loop of the aminoacyl-site tRNA in the 30S subunit. This blocks tRNA into the A-site tRNA, preventing protein synthesis and bacterial replication [44]. The topical agent also appeared to maintain the favorable resistant profile of oral minocycline, with the frequency of spontaneous resistance to topical minocycline being as low as ≤1 × 10^−8^ in *C. acnes* strains [43].

Dermal safety studies of topical minocycline did not show evidence of phototoxicity or photoallergic responses; however, it is recommended that patients undergoing treatment minimize or avoid exposure to natural or artificial sunlight. The most common adverse events associated with topical minocycline 4% and 1.5% foam are headache and diarrhea, respectively. Patients who use topical minocycline may also experience a temporary yellow “glare” on the skin due to the yellow tint of the minocycline molecule contained in the preparation [45]. This is not considered to be a staining of the skin and can be washed off with soap and water after waiting at least 1 h after application [45]. Applying topical minocycline as part of a pre-bedtime routine may also help alleviate concerns associated with yellow discoloration of fabrics.

### 3.10. Mupirocin

Mupirocin is a topical antibacterial agent supplied as a 2% ointment or cream. Mupirocin is a naturally occurring antibiotic isolated from the bacterium *Pseudomonas fluorescens* NCIMB 10586 fermentation broth, being produced as mixture of four pseudomonic acids (A, B, C, D) with a basic structure of a monic acid containing a pyran ring [46]. Pseudomonic acid A represents the main compound of mupirocin. Mupirocin displays a broad-spectrum activity against Gram-positive (*S. aureus* and *Strep. pyogenes*) and certain Gram-negative bacteria.

Mupirocin is known to be bacteriostatic at low concentrations and bactericidal at high concentrations [46]. Mupirocin works by inhibiting the enzyme isoleucyl-tRNA synthetase via competitive inhibition. Binding to the enzyme prevents the conversion of isoleucine to isoleucine-charged transfer RNA, causing cellular depletion of isoleucine-charged transfer RNA. This eventually leads to the cessation of protein and RNA synthesis, resulting in bacterial death. Mupirocin is commonly used for treating skin and skin structure infections, including penicillin-resistant staphylococcus aureus (MRSA) cutaneous infections or nasal decolonization to prevent transmission and infection. Mupirocin is indicated for the treatment of impetigo and secondarily infected superficial cutaneous wounds due to *Staphylococcus aureus* and *Streptococcus pyogenes* in adults and children [47]. For the treatment of bullous and non-bullous impetigo, mupirocin or retapamulin twice daily (bid) for 5 days can be considered [1]. For staph decolonization, mupirocin can be administrated topically to the skin or nares. Once applied, mupirocin is rapidly converted into inactive monic acid and is excreted through the kidneys [46]. The elimination half-life is 20–40 min for mupirocin and 30–80 min for monic acid.

Since the introduction of mupirocin to the market in 1987, there have been varying reports of MRSA resistance ranging from 1–81% worldwide [46]. There are many factors that are driving bacterial resistance to mupirocin, one being its availability as an over-the-counter drug in certain countries.

In the United States, resistance is attributed to a number of factors. The incidence of community-associated skin and soft-tissue infections have increased in recent years, and in an effort to prevent recurrence of infections, many clinicians are increasing the use of mupirocin to eradicate MRSA colonization. Another factor is the growing interest in perioperative eradication of *S. aureus* colonization as a strategy for preventing post-surgical infection [48]. There is an emerging body of evidence suggesting that perioperative eradication of *S. aureus* can reduce the number of postsurgical staphylococcal infections, and thus will likely lead to increased use of mupirocin [48].

Mupirocin resistance can also be attributed to the development of bacterial genes that provide micro-organisms protection from mupirocin’s mechanism of action, especially in *S. aureus*. The categories of mupirocin susceptibility have been described for *S. aureus* based on minimum inhibitory concentrations (MICs). The three categories include *S. aureus* with a MIC of ≤4 µg/mL, low-level mupirocin resistance with MICs from 8 to 64 µg/mL, and high-level mupirocin resistance with MICs ≥ 512 µg/mL [48]. Studies have shown that *S. aureus* with high-level mupirocin resistance have acquired a plasma-mediated *mupA* gene, which encodes for a novel isoleucyl RNA synthetase, disrupting the activity of mupirocin [46,48]. Isolates with low-level mupirocin resistance have base changes on the native isoleucyl RNA synthetase gene, *ileS* [48]. The mechanisms of mupirocin resistance by *S. aureus* demonstrates the emergence of increased drug resistance and the need to monitor overuse of topical mupirocin in patients.

The overuse of mupirocin can lead to the development of drug-resistant microbes, resulting in suboptimal patient outcomes from inadequate response to therapy. It is important that dermatologists incorporate principles of antibiotic stewardship to help reduce antimicrobial resistance. Strategies primarily focus on prescribing the optimal dose and shortest effective duration of antibiotic treatment, and using nonantibiotic therapies when possible (e.g., considering no prophylactic antibiotics for low-risk groups, considering topical decolonization with antiseptic with chlorhexidine, or the use of intra-incisional antibiotics for surgical site infection prophylaxis) [49]. Such steps can help optimize appropriate antibiotic use, but further studies that evaluate the unintended consequences of mupirocin use as a prevention strategy, monitoring the prevalence of resistance where mupirocin is commonly used, and the development of mupirocin susceptibility testing to guide therapeutic use of mupirocin, are all warranted [48].

The adverse events of mupirocin for all age groups are typically mild and include, but are not limited to, headache, burning, stinging, or pain in the affected area being treated, itching, rash, or nausea. Contraindications of topical mupirocin use include patients with known hypersensitivity to mupirocin or any of the excipients of mupirocin cream.

### 3.11. Neomycin

Topical neomycin is a bactericidal, aminoglycoside antibiotic that acts by binding to the 30S subunit of the bacterial ribosome inhibiting bacterial protein synthesis. [2,9]. Topical neomycin has been shown to have excellent activity against Gram-negative bacteria such as *E. coli*, *H. influenzae*, proteus, and serratia, but has partial activity against Gram-positive bacteria [2,9]. Topical neomycin has the ability to kill staphylococci, but has weak activity against *Streptococci* [9].

As a topical cream or ointment that is available over the counter, topical neomycin is commonly formulated with bacitracin (Gram-positive coverage) and polymyxin (Pseudomonal coverage). Bacitracin is produced by *Bacillus subtilis* and is composed of a congregation of cyclic polypeptide antibiotics [50]. The mechanism of action of bacitracin relies on its ability to prevent the transfer of mucopeptides into the cell walls of bacteria, inhibiting bacterial cell wall synthesis and replication [50]. These actions eventually lead to bacterial cell death. Bacitracin is shown to have both bacteriostatic and bactericidal properties depending on drug concentration [50]. Bacitracin has activity against Gram-positive bacteria, but most Gram-negative bacteria are known to be resistant. Polymyxins are obtained from *Bacillus polymyxa* and possess bactericidal activity against Gram-negative bacteria, but have poor activity against Gram-positive bacteria [2]. Polymyxins act on the outer membrane of Gram-negative bacteria by interfering with the micro-organism’s phospholipids [51]. This destabilization alters the structure of the cell membrane, leading to increased permeability and leakage of intracellular contents, causing bacterial death [51]. The triple formulation, consisting of neomycin–bacitracin–polymyxin, is commonly used to treat minor wounds, secondarily infected dermatitis, and superficial pyodermas [9]. The most common adverse events associated with the triple formulation include erythema, itching, swelling, irritation, or the development of allergic contact dermatitis at the affected site. Patients with hypersensitivity to any of the components of the formulation should avoid its use.

### 3.12. Ozenoxacin

Topical ozenoxacin cream 1% is a non-fluorinated quinolone antibiotic that predominantly executes its function by inhibition of bacterial DNA replication enzymes, DNA gyrase A and topoisomerase IV [52]. Ozenoxacin was FDA-approved in 2017 for the treatment of impetigo caused by *S. aureus* or *S. pyogenes* in patients 2 months of age and older [53]. Ozenoxacin has been shown to be bactericidal against *S. aureus* and *S. pyogenes*. Due to increasing antibiotic resistance with the use of other topical antibiotics, ozenoxacin can be considered as an alternative since it has negligible systemic absorption and expanded spectrum against methicillin-, mupirocin-, and ciprofloxacin-resistant strains of *S. aureus* [53]. Regarding quinolone resistance for other micro-organisms, resistance can potentially arise through mutations of genes that encode for DNA gyrase or topoisomerase IV. Resistant organisms typically carry a combination of mutations with *gyrA* and *parC* subunits [54]. Despite the possibility of the development of mutations, ozenoxacin is still considered a secure alternative, since the overall frequency of resistant mutants selected by ozenoxacin is ≤10^−10^ [54].

### 3.13. Retapamulin

Topical retapamulin is a novel topical semisynthetic antibacterial of the pleuromutilin class. Pleuromutilins are a class of antibiotics discovered in the 1950s by the isolation of the naturally occurring pleuromutilin from *Pleurotus mutilus* (now renamed *Clitopilus scyphoides*), an edible mushroom [55]. Retapamulin has a bacteriostatic mechanism of action that is similar to that of macrolides and clindamycin by selectively inhibiting the initiation of protein synthesis in bacterial 50S ribosomes. By binding to the ribosomal P site of the 50S ribosome, pleuromutilins inhibit peptidyl transferase, block P-site interactions, and prevent the formation of 50S ribosomal subunits.

**Figure 1:**
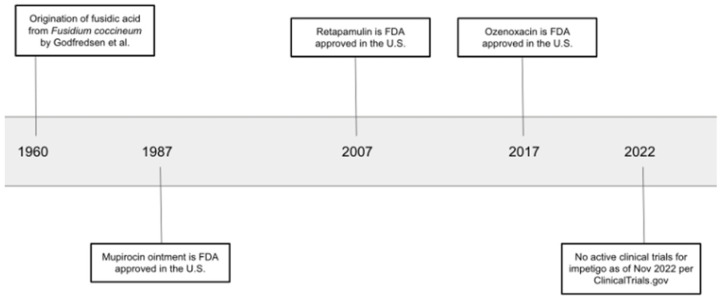
Timeline of discovery and approval of topical antibiotics for impetigo [55,56,57].

Retapamulin 1% ointment was approved by the FDA in 2007 for the treatment of impetigo caused by methicillin-susceptible *S. aureus* and *S. pyogenes* in adults and children older than nine months (Figure 1) [56]. Currently, there is a lack of efficacy data and clinical evidence to support the use of retapamulin for methicillin-resistant *Staph. aureus* (MRSA), especially mupirocin-resistant strains. An in vitro study evaluating the susceptibility of mupirocin-resistant MRSA, mediated by the plasmid-borne *mupA* gene, demonstrated that all isolates collected in the study from pediatric patients were susceptible to retapamulin. The data revealed that retapamulin may be a promising alternative for such strains, but further research is needed to evaluate the in vivo efficacy of retapamulin to support its use as a topical agent for MRSA and mupirocin-resistant MRSA strains [57].

### 3.14. Silver Sulfadiazine

Silver sulfadiazine is a topical sulfonamide antibiotic that acts on the cell membrane and cell wall to produce its bactericidal effect. The medication is available as a water-soluble cream at a strength of 1%. Silver sulfadiazine is available as both a prescription and as an over-the-counter wound-care product. The specific mechanism of how this antibiotic functions has not been completely understood, but it is suggested that the silver ions bind to cellular proteins and the surface membrane, leading to protein denaturation and proton leak in the membrane. The sulfadiazine component acts as an inhibitor of folic acid synthesis. These two components act synergistically, leading to bacterial death. This topical antibacterial is most commonly used to treat or prevent serious infections on areas of the skin in patients with second- or third-degree burns [9]. The medication can also be used for superficial skin infections, cellulitis, ulcerations, and ecthyma gangrenosum. The drug should be avoided in patients with known or suspected sulfonamide allergy or hypersensitivity to any of its components.

## 4. Conclusions

The skin is the first line of defense against many micro-organisms, but when compromised, the need for topical antibiotics arises in dermatology. Based on clinical judgment and evidence-based medical practice, dermatologists should strategically use topical antibiotics, keeping in mind the indication, the location of dermatologic concern, and emergence of drug resistance (Table 1 and Table 2).

## 5. Future Directions

Topical antibiotics may alter the cutaneous microbiome, and with prolonged exposure can change the bacteriology of the nares and oropharynx. In the future, this can lead to the emergence of resistant pathogens and warrants judicious decision-making by dermatologists when prescribing topical antibiotics. Further research is also needed on cross-reactivity and contact allergies to topical antibiotics to lower the rate of contact dermatitis induced by these medications. There have been many in vitro studies investing botanical products against skin micro-organisms, but further large scale randomized clinical trials would be beneficial to establish clinical applications of these products [9].

## Figures and Tables

**Table 1:** Topical antibiotics and common uses.

Acne Vulgaris	Rosacea	Hidradenitis Suppurativa	Secondarily-Infected Dermatitis	Skin and Soft-Tissue Infection (ex: Impetigo)
Azelaic Acid	Azelaic acid	Clindamycin	Fusidic Acid	Amikacin
Benzoyl Peroxide	Minocycline		Mupirocin	Clindamycin
Clindamycin	Metronidazole			Fusidic acid
Dapsone				Mupirocin
Minocycline				Ozenoxacin
				Retapamulin

**Table 2:** Characteristics of topical antibiotics.

Brand Name	Generic Name	Preparation	Dosage	Approved Indications	Age for Use	Bacterial Activity
Azelex; Finacea	Azelaic Acid	Cream, Foam, Gel	15% (Gel/Foam) 20% (Cream)	Acne Vulgaris, Rosacea	≥12 years	Gram +
Benzac; Benzefoam; Benzepro; Clean and Clear; Clearasil	Benzoyl Peroxide	Cream, Foam, Gel, Solution, Lotion	2.5–10%	Acne Vulgaris	≥7 years	Gram +
Cleocin; Cleocin-T; Clindacin ETZ; Clindacin Pac; Clindagel; Clindesse; Evoclin	Clindamycin	Cream, Foam, Gel, Lotion, Solution, Swab	1%, 2%	Acne Vulgaris	≥12 years	Gram +/−
Aczone	Dapsone	Gel	5%, 7.5%	Acne Vulgaris	≥9 years (Gel 7.5%) ≥12 years (Gel 5%)	Gram +/−
Fucidin (Available in Canada; not available in the US)	Fusidic Acid	Cream, Ointment	2%	Skin/Soft Tissue Infections	≥5 years	Gram +
Garamycin	Gentamicin	Cream, Ointment	0.1%	Primary/Secondary Skin Infections	≥1 year	Gram −
MetroCream; Metrogel; MetroLotion; Noritate; Rosadan	Metronidazole	Cream, Gel, Lotion	0.75%, 1%	Rosacea	≥18 years	Anaerobes
Amzeeq; Zilxi	Minocycline	Foam	1.5%, 4%	Acne Vulgaris, Rosacea	≥18 years (Foam 1.5%) ≥9 years (Foam 4%)	Gram +/−
Bactroban; Centany; Pirnuo	Mupirocin	Cream, Ointment	2%	Impetigo, Secondarily Infected Skin Lesions	≥2 months	Gram +
Neosporin; Triple Antibiotic	Neomycin/Bacitracin/ Polymyxin B	Cream, Ointment	0.5%	Secondary Skin Infections	≥2 years	Gram −
Xepi	Ozenoxacin	Cream	1%	Impetigo	≥2 months	Gram + > Gram −
Altabax; Altargo	Retapamulin	Ointment	1%	Impetigo	≥9 months	Gram +
Silvadene; SSD; Thermazene	Silver Sulfadiazine	Cream	1%	Prevention/Treatment of Wound Sepsis in Second- and Third-Degree Burns	>2 months	Gram +/−

## Data Availability

The authors confirm that the data supporting the findings of this study are available within the article.
